# Iron Fortification Practices and Implications for Iron Addition to Salt

**DOI:** 10.1093/jn/nxaa175

**Published:** 2021-02-15

**Authors:** Richard F Hurrell

**Affiliations:** Institute of Food, Nutrition and Health, ETH Zurich, Zurich, Switzerland

**Keywords:** iron fortification, iron fortification vehicles, iron fortification technologies, iron fortification compounds, iron bioavailability, sensory changes

## Abstract

This introductory article provides an in-depth technical background for iron fortification, and thus introduces a series of articles in this supplement designed to present the current evidence on the fortification of salt with both iodine and iron, that is, double-fortified salt (DFS). This article reviews our current knowledge of the causes and consequences of iron deficiency and anemia and then, with the aim of assisting the comparison between DFS and other common iron-fortified staple foods, discusses the factors influencing the efficacy of iron-fortified foods. This includes the dietary and physiological factors influencing iron absorption; the choice of an iron compound and the fortification technology that will ensure the necessary iron absorption with no sensory changes; encapsulation of iron fortification compounds to prevent unacceptable sensory changes; the addition of iron absorption enhancers; the estimation of the iron fortification level for each vehicle based on iron requirements and consumption patterns; and the iron status biomarkers that are needed to demonstrate improved iron status in populations regularly consuming the iron-fortified food.

The supplement is designed to provide a summary of evidence to date that can help advise policy makers considering DFS as an intervention to address the difficult public health issue of iron deficiency anemia, while at the same time using DFS to target iodine deficiency.

## Introduction

Food fortification has an impressive history of public health successes (1, 2), and national fortification programs have helped eliminate, or greatly decrease, many of the micronutrient deficiencies that were common in Europe and the United States at the beginning of the 20th century. Wheat flour fortification programs with iron were introduced nationally in United States and the United Kingdom in the 1940s so as to target widespread anemia. Iron fortification of wheat flour is now mandatory in 81 countries and, when designed, implemented, and monitored correctly, would be expected to improve or maintain an adequate iron status (3).

Although iron fortification of wheat flour in some low- and middle-income countries (LMIC) has resulted in only modest decreases in anemia prevalence (4), this is not unexpected because iron deficiency (ID) is estimated to account globally for only ∼50% of anemia (5), and ID makes an even lower contribution to anemia in many LMIC (6), where infections and inflammation, hemoglobinopathies, hookworm, and other nutritional deficiencies are often more important causes of anemia than ID (7). Hemoglobin (Hb) alone therefore must not be used to monitor iron interventions. When iron interventions are monitored by specific biomarkers of iron status, such as serum ferritin, there is ample evidence that regular consumption of iron-fortified foods markedly improves iron status (8–10).

Nevertheless, the development of efficacious iron-fortified foods has proven more challenging than developing nutritionally efficacious foods fortified with other micronutrients. This is because, unlike most other micronutrients, iron can cause unacceptable sensory changes to foods. When added to certain foods (including salt), the more bioavailable, soluble iron compounds cause unacceptable color and flavor changes, whereas the less soluble iron compounds cause less sensory changes in the double-fortified salt (DFS) and cooked foods, but are much less well absorbed. Although these problems have now been largely overcome, and efficacious, sensorily acceptable iron fortification technologies have been developed for most major food fortification vehicles (9), some challenges remain including preventing color changes in salt.

This introduction is the first of a series of background articles for the DFS global consultation and is followed by a series of articles with a more specific focus on DFS as a vehicle for iron fortification. The series progresses from an in-depth discussion of the technology required for producing DFS, to a statistical review of efficacy and effectiveness, to a review of global programs where DFS has been piloted, initiated, or implemented, and finally to a comparison of DFS with the more established iron fortification efforts using other vehicles.

## Prevalence, Causes, and Consequences of ID and Anemia

Women, children, and female adolescents in LMIC are most at risk of ID and anemia (11). Insufficient dietary iron intake results in a progressive decline in Hb concentrations in RBCs, leading to iron deficiency anemia (IDA) and a lower ability of the blood to carry oxygen. ID is caused by inadequate iron intake, poor iron bioavailability, restricted iron utilization, and/or by excessive iron losses. The common diet in many LMIC mostly contains poorly bioavailable iron from plant foods and little or none of the more bioavailable iron from animal tissue foods.

Anemia affects an estimated 1.62 billion people globally, mainly in LMIC, but also to a lesser extent in high-income countries (12). Anemia prevalence varies widely with socioeconomic status and geographical location. In 2013, anemia prevalence for children aged <5 y varied from 11% in high-income countries to ≤50–70% in the LMIC of South Asia and sub-Saharan Africa. For women of childbearing age, anemia prevalence varied from 16% in high-income countries to almost 50% in countries in South Asia, Central Africa, and West Africa (13). The global prevalence of ID, however, is not known with precision, because in many countries iron status has not always been measured specifically but estimated based on anemia prevalence, by assuming 50% of anemia is IDA. This is far from precise, and the exact proportion of anemia caused by ID varies considerably depending on the region of the world and the characteristics of the population (7).

Whereas the cause of anemia in high-income countries is more likely to be ID, widespread infections, inflammation, and hemoglobinopathies play a much greater role in LMIC. Petry et al. (6) estimated that ID accounted for only 25% of anemia in children and 37% of anemia in women of reproductive age living in LMIC with widespread infections. Kassebaum et al. (5) listed 17 causes of anemia and, although low iron intake or absorption were the most common causes (50%), other major causes were inflammation caused by infections such as malaria, HIV, and tuberculosis; blood loss caused by parasites such as hookworm; hemoglobinopathies such as sickle-cell disease and thalassemia; and other nutritional deficiencies such as vitamin B-12, folate, and vitamin A, all of which are more common in LMIC (5) than in high-income countries. Where malaria infection is endemic, a restriction in the recycling of red cell iron in infected subjects, increased lysis of infected red cells, and an inflammation-related inhibition of iron absorption, are more important causes of anemia than low iron intake (14).

Estimating ID prevalence based on anemia is thus imprecise and unreliable and should not be used to evaluate the need for iron fortification. Additionally, as mentioned earlier, anemia prevalence alone cannot be used to monitor iron fortification interventions because the additional iron will only impact on the proportion of anemia resulting from ID and not on anemia resulting from other causes. The perceived modest impact of some large-scale iron fortification programs with wheat flour could have been due in part to using anemia prevalence to monitor program performance where infections, inflammation, and hemoglobinopathies are major contributors to anemia (4).

The negative health consequences of ID and IDA, caused by low dietary iron absorption or infection/inflammation, include poor brain development in the fetus and young child, poor cognitive function in children, poor iodine utilization, reduced work capacity, poor pregnancy outcomes, and decreased survival of a child born with low iron status (15). However, the difference in negative health consequences caused by IDA and other anemias is not clear. Iron is needed for fetal brain development, iodine utilization, and energy-producing enzymes (16); however, anemia without ID would still decrease work capacity due to lack of oxygen and could also result in poor pregnancy outcomes, including increased maternal mortality, low birth weight, preterm birth, and still births (17).

## Dietary Components that Influence Iron Absorption

Iron-fortified foods such as cereal flours, rice, salt, or milk are all consumed as part of a mixed diet. Within that regular mixed diet, and within the food fortification vehicle itself, there are food constituents that can have a major impact, both positive and negative, on the absorption of iron fortification compounds, as well as on the absorption of native food iron. The iron bioavailability from the regular mixed diet to which the fortified food is to be added has a major influence on the design of the iron fortification program. It is used to ensure that the selected fortification level, and the additional amount of fortification iron absorbed, are sufficient to fill the gap between the amount of iron currently consumed and the amount of absorbed iron needed to achieve adequate iron status.

The main dietary inhibitors of iron absorption are phytic acid and polyphenol compounds. Phytic acid occurs at especially high concentrations in cereal grains, including wheat, maize, and rice (18), and in legume seeds such as common beans, lentils, and soy (19, 20). Polyphenol compounds are mostly consumed via beverages such as tea, coffee, and cocoa (21), but are also found in relatively high quantities in some vegetables, fruits, colored beans, spices, and sorghum varieties (22–24). Fortification vehicles, such as wheat flour, maize flour, and rice, contain sufficient phytic acid to substantially decrease iron absorption (18, 25). Phytic acid is mainly in the cereal bran (26), and wholegrain products are the most inhibitory. Removal of the bran during milling of wheat and maize flour or polishing of rice, can substantially increase iron absorption. Iron absorption from bread rolls made with white wheat flour was reported to be 6 times higher than from wholegrain wheat flour (27). Other dietary inhibitors include calcium from milk products and certain proteins from milk and legumes (28).

In contrast, ascorbic acid from fruits and vegetables (29, 30) and peptides from partially digested muscle tissues from meat, fish, and poultry (31) enhance iron absorption and can overcome to some extent the negative effects of phytic acid and polyphenols. Iron bioavailability from a diet thus depends on the balance between the inhibitors and enhancers of iron absorption. Typical diets from LMIC would be expected to be high in phytic acid and low in ascorbic acid and animal foods. WHO/FAO (32) propose 5–15% iron bioavailability depending on diet composition, with the lowest bioavailability of 5% being for diets based on cereals and/or tubers with negligible amounts of muscle tissue and ascorbic acid. Hallberg and Rossander-Hultén (33) estimated iron bioavailability from omnivore Western diets to be 14–17% compared with 5–12% from Western vegetarian diets. This compares with an estimate of 7.5–13.4% for the iron bioavailability of typical Latin American diets in the 1980s (34). The expected dietary iron absorption from the iron-fortified foods would reflect the iron bioavailability of the whole diet and not the iron bioavailability from the fortified food vehicle alone.

It might seem surprising that despite the high levels of iron absorption inhibitors, and the low levels of food components that enhance iron absorption in the diets of many LMIC, iron-fortified foods still improve iron status. For example, in DFS studies in Morocco, the common diet consumed by schoolchildren was based on wheat bread, couscous, fava beans, and chickpea, and was reported to be high in phytic acid and low in ascorbic acid and animal tissue foods (35). Nevertheless, providing the children with fortification iron via DFS, either as encapsulated ferrous sulfate (35) or as ferric pyrophosphate (36), decreased the prevalence of IDA in the schoolchildren from >30% at baseline to <10% at the end of the studies. The prevalence of IDA increased again to 33% 15 mo after the children had returned to their normal diet, which included iodized salt but no DFS (37).

These Moroccan children consumed a diet that was highly inhibitory to iron absorption, so the likely explanation for the above results is that the fractional iron absorption in the children, although low, was sufficient to meet their iron needs when they consumed the additional fortification iron in DFS but not when they consumed salt without added iron. Low iron stores in children with ID would lower hepcidin production by the liver and enhance fractional iron absorption from the low bioavailability diet (38).

## Factors Influencing the Choice of the Iron Fortification Compound

A large number of iron compounds have been considered for the fortification of foods. Allen et al. (39) listed 19 iron compounds together with their relative absorption in humans compared with ferrous sulfate as well as their cost relative to ferrous sulfate. The iron compound selected for food fortification is usually the most bioavailable compound that causes no sensory changes to the food vehicle at an acceptable cost (9). The influence of added iron on the stability of other nutrients can also be an issue and, in relation to DFS, the fortification iron compound should have little or no influence on the stability of iodine. In practice, a small number of iron compounds are more regularly used for food fortification. These include ferrous sulfate, ferrous gluconate, ferrous fumarate, ferric pyrophosphate (FPP), sodium iron ethylenediaminetetraacetic acid (NaFeEDTA), ferrous bisglycinate (FBG), and elemental iron powders.

### Relative bioavailability

The relative bioavailability of different iron fortification compounds has been discussed in detail by Hurrell (9). In summary, iron from ferrous sulfate added to a food is absorbed to the same extent as native food iron because it is influenced in the same way by inhibitory and enhancing food components and by the physiological state of the consumer. The relative absorption of other iron compounds is governed by the extent to which they dissolve in the gastric fluid during digestion. Ferrous sulfate and ferrous gluconate are water soluble and dissolve readily in the gastric fluid. Ferrous fumarate is poorly water soluble but dissolves completely in the dilute acid of the gastric fluid during digestion, and is considered to have the same bioavailability as ferrous sulfate. FPP and elemental iron powders only partially dissolve in the gastric fluid and their relative absorption is governed by their extent of dissolution. NaFeEDTA and FBG are iron chelates that have the same bioavailability as ferrous sulfate in the absence of iron absorption inhibitors but a 2–4-fold higher absorption in their presence.

Iron absorption is also regulated with respect to an individual's iron status with the aim to ensure sufficient iron absorption to meet iron needs while avoiding excess iron absorption that could lead to negative health consequences (9). Thus, an individual with low iron status markedly increases iron absorption so as to cover the additional needs, whereas an individual with adequate iron status decreases absorption as necessary to cover the lower requirements. Iron absorption from soluble iron compounds (such as ferrous sulfate) is more efficiently upregulated with low iron status than absorption from more insoluble compounds, such as FPP. The relative absorption of FPP is thus lower in individuals with ID than in individuals with adequate iron status (40).

### Sensory changes compared with relative bioavailability

**FIGURE 1:**
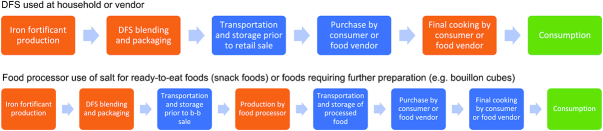
Two potential product and distribution pathways for double-fortified salt (DFS). When evaluating opportunity for a new food vehicle or the addition of a new nutrient to an existing food vehicle, research can be necessary to consider any potential for nutrient interactions, nutrient losses, or sensory changes (e.g., changes in color, taste, smell) at various points in a product's pathway. Using DFS as an example (depending on the iron formulation or compound used), these could occur at production or processing steps (in orange) or storage and transportation (in blue). Whether these changes significantly affect the product from an iodine retention or consumer acceptance standpoint are realized at the consumption stage (in green). b-b, business-to-business.

Although adequate iron bioavailability of an iron compound is needed to ensure efficacy, a more important consideration for the food manufacturer in the selection of an iron compound is the extent to which it might interact with the food vehicle and lead to adverse changes in taste and/or color that might render the fortified product undesirable to consumers. Several commonly fortified food vehicles are not just consumed directly but are also ingredients in processed foods (e.g., salt, flours, oil). Processed foods typically go through a longer product pathway before reaching consumers, which can include cooking and/or several storage periods along its distribution process. **Figure 1** presents 2 potential pathways for DFS. As a result, there are several points along a food vehicle's production and use pathway where there can be exposure to potential interactions that lead to sensory and nutrient retention changes, not just to the fortified food, but also to a food processed with fortified ingredients.

#### Water-soluble iron compounds

Based on bioavailability and cost, ferrous sulfate would usually be the first choice for iron fortification. Unfortunately, water-soluble iron compounds are the most likely to cause unacceptable sensory changes to the food fortification vehicles or the foods they are consumed with (9). When added to foods containing phenolic compounds, ferrous sulfate is liable to cause unacceptable blue or brown coloration, and in liquid products can give a metallic taste. Addition of ferrous sulfate to some cereal flours, especially those stored under hot, humid conditions, can also provoke lipid rancidity. The iron chelates NaFeEDTA and FBG are also water soluble and can cause similar unacceptable sensory changes to food fortification vehicles.

#### Water-insoluble compounds, readily soluble in the gastric fluid

Ferrous fumarate has the same bioavailability as ferrous sulfate but causes far less sensory changes. It is widely used to fortify cereal-based complementary foods (41).

#### Compounds partly soluble in the gastric fluid

FPP and elemental iron powders are the preferred iron compounds for foods that are highly sensitive to unacceptable color and flavor changes, because these compounds cause few if any sensory changes. However, they are insoluble in water and only partially dissolve in the gastric fluid during digestion, so have a lower fractional iron absorption than ferrous sulfate or ferrous fumarate. However, this lower relative bioavailability is overcome by adding these compounds at a higher fortification level so as to achieve the same amount of total iron absorbed.

FPP is about half as well absorbed as ferrous sulfate so must be added to foods at double the iron concentration (39). It is frequently used by the food industry for fortification of food products that are liable to form unacceptable colors with other iron compounds, such as infant cereals containing fruits or vegetables, chocolate drink powders, and bouillon cubes (9, 42). FPP is reported to be the only compound that does not discolor extruded rice kernels (43) and, in the DFS storage studies of Wegmüller et al. (44), was one of the few iron compounds that did not discolor DFS. Three forms of FPP are available commercially. These are regular FPP with a mean particle size of 20 μm, micronized ground FPP with a mean particle size of 2.5 μm, and micronized dispersible FPP, which is an agglomerate of extremely small FPP particles (0.3 μm) and emulsifiers, and is dispersible in liquid products.

Iron powders are the least expensive iron compound and are commonly used to fortify wheat flour in the United States, with many LMIC initially following the US example. There are 5 different types of elemental iron powder depending on the manufacturing process, and the relative bioavailability of these powders is reported to vary widely (45). Reduced iron powders (H-reduced or atomized) have been the most widely used to fortify cereal flours; however, based on evidence from absorption and efficacy studies, electrolytic iron powder is the only iron powder that is recommended for food fortification by WHO (45). It is assumed to be 50% as well absorbed as ferrous sulfate and recommended to be added at double the iron fortification level as used for ferrous sulfate (39).

#### Encapsulated compounds

Encapsulation technologies have been developed to maintain the higher bioavailability of an iron fortification compound without causing adverse sensory changes. For example, encapsulation of ferrous sulfate and ferrous fumarate with partially hydrogenated lipids can prevent or decrease color and flavor changes in sensitive foods without any apparent decrease in bioavailability, and have been recommended by WHO (39). The most common capsular material is hydrogenated soybean oil, with a melting point of 65°C. This capsule appears to be digested by both humans and rats and the iron compounds suitably released for absorption. In animal studies, iron bioavailability from ferrous sulfate or fumarate appears to be little affected by the encapsulation provided that the capsule is ≤ ∼50% of the total compound (46); good iron efficacy with a ∼50/50 iron/capsule ratio has been reported in human studies (35, 47).

The University of Toronto and Nutrition International (NI, formerly The Micronutrient Initiative) developed an encapsulated ferrous fumarate (EFF) specifically for use in DFS. The capsule is more sophisticated and refined than the partially hydrogenated oil capsule and includes soy stearine, titanium dioxide (to mask the red color of ferrous fumarate), hydroxy propyl methyl cellulose (HPMC), and sodium hexametaphosphate (SHMP), and represents ∼55% of the compound. The influence of this more sophisticated capsule on iron bioavailability from ferrous fumarate has not been measured in either animals or humans. The capsule must be digested, and the ferrous fumarate released into the gastric fluid early enough for the iron to be completely dissolved during the time the food remains in the stomach. If the iron is not completely released and dissolved in the gastric fluid, the relative bioavailability of ferrous fumarate will be decreased.

#### Iron chelates

When the food fortification vehicle, or the regular diet, is high in phytic acid or other iron absorption inhibitors, the best option to enhance iron absorption from the fortified staples and condiments is the addition of NaFeEDTA or FBG. NaFeEDTA overcomes the inhibitory effect of phytic acid on iron absorption and to a lesser extent overcomes the inhibitory nature of polyphenol compounds (9). NaFeEDTA increases iron absorption 2–4-fold from cereal- and legume-based foods (48). Native food iron absorption is likewise increased on consumption of an NaFeEDTA-fortified food with an inhibitory meal; however, NaFeEDTA will not increase iron absorption from noninhibitory meals (49). NaFeEDTA is also recommended for soy and fish sauces, where it prevents iron-initiated precipitation of peptides; NaFeEDTA-fortified fish sauce has effectively improved iron status in Vietnamese women fed rice meals (50), and NaFeEDTA-fortified soy sauce has improved the iron status of Chinese adults (51).

FBG, like NaFeEDTA, overcomes phytic acid inhibition of iron absorption and has a moderate enhancing effect on iron absorption in the presence of phenolic compounds. There are several studies demonstrating its efficacy when added to liquid milk, yoghurts, and other dairy products in Latin America (52). It is currently used to fortify liquid milk, powdered milk, and maize flour in the successful national fortification program in Costa Rica (53). Other iron glycine chelates exist but have been much less investigated than FBG, which is a patented compound.

### Cost

Allen et al. (39) gave indicative costs of the different iron compounds per milligram iron relative to ferrous sulfate at a relative cost of 1. Current relative costs appear little changed, with electrolytic iron at 0.4, ferrous fumarate 1.9, FPP 3.6, micronized ground FPP 4.6, ferrous gluconate 5.2, NaFeEDTA 13, and FBG 13 (K Brockhausen, Dr. Paul Lohmann GmbH & Co KGaA, personal communication, 2019). Ferrous sulfate and ferrous fumarate encapsulated with partially hydrogenated palm oil are currently 8.7 and 7.3 times, respectively, the cost of nonencapsulated ferrous sulfate. The cost of NI EFF relative to ferrous sulfate has not been reported.

## Fortification Technologies, and Relevant Iron Fortification Compounds, for Major Food Fortification Vehicles

### Product development for fortifying new food vehicles or adding nutrients to existing food vehicles

The purpose of fortifying staple foods is to improve micronutrient intake through foods already consumed within the diet. Consumers are already familiar with the nonfortified varieties of these staple foods, hence a key tenet of food fortification is to avoid any color, taste, or odor changes to the fortified food that might cause rejection. Any micronutrients added to a food must also be retained in the final food when it reaches the consumer—if not there is no public health benefit to fortification.

**Textbox 1** lays out a typical product development pathway to verify that *1*) fortification does not cause undesirable sensory changes to a food vehicle *and* any foods prepared with that food as an ingredient, and *2*) micronutrients added to the food vehicle are bioavailable when consumed (efficacy).

Textbox 1 Steps needed for the development of a new iron-fortified food for introduction into a national fortification program.

1. **Select food fortification vehicle** based on national consumption patterns and estimate fortification level based on i) iron requirement (mg/d) of most at-risk group in the population (usually women of child-bearing age), and ii) their food vehicle consumption pattern. The iron requirement value used to estimate the fortification level must be based on the estimated iron bioavailability of the regular diet in the country concerned. For low -and middle-income countries, this is usually 5–10% compared with 15–18% for high-income countries.
2. After the food vehicle is selected, the next step is to **select the iron fortification compound** based on sensory studies, relative bioavailability, and cost. All major iron fortification compounds have been tested for relative bioavailability in humans using radio or stable isotopes. Perform sensory studies (color, flavor, texture) with the iron compound of highest relative bioavailability and lowest cost (usually ferrous sulfate), added to the food vehicle during processing, storage, and preparation for consumption. If the iron compound is unacceptable, then evaluate the sensory properties of a less soluble compound such as ferrous fumarate, and if this is still unacceptable, evaluate the more insoluble FPP or electrolytic iron, which have lower bioavailability but cause little or no sensory changes. If the latter compounds are finally selected as the iron fortification compound, they must be added at a higher fortification level based on their relative bioavailability as measured in human studies. Usually, the first sensory studies would be a simple screening, and more elaborate sensory studies would be made with the selected compound(s).
3. If a novel iron compound is developed, or an existing iron compound technically modified, such as by encapsulation, this compound should be **tested for relative bioavailability**. Relative bioavailability can be tested first in rat assays (Hb repletion) before making human studies with isotopically labeled compounds. Sometimes, such as with elemental iron powders or encapsulated iron compounds, it is difficult to get an isotopically labeled iron compound of exactly the same physical and chemical characteristics as the commercial compound.
4. In order to more precisely define the fortification level of all iron compounds, iron absorption in humans from the iron-fortified food alone, and from the iron-fortified food combined in traditional meals can be made. **Multimeal stable isotope studies** give a better estimation of iron bioavailability from the regular diet containing the iron-fortified food.
5. If the fractional iron absorption from the iron-fortified food is low, because of the high concentration of phytic acid, such as in whole cereals, or if the food contains other inhibitors of iron absorption, such as calcium in milk products, it might be possible to add an absorption enhancer. Ascorbic acid addition can be used to enhance the absorption of the fortification iron, or NaFeEDTA or ferrous bisglycinate can be considered as the iron fortification compound. **Sensory studies need to be made and the stability of ascorbic acid during processing and storage needs to be ensured**.
6. **Nutrient retention studies** during processing and storage can be made at this stage (or earlier). Although iron itself is not sensitive to processing or storage losses, the addition of iron can increase the losses of other nutrients, such as iodine or retinol, that are sensitive to losses during processing and storage.
7. More elaborate **sensory studies** with the selected iron compound will be needed at some stage to carefully check for any changes due to processing or storage of the food vehicle, or to processed foods to which the fortified food (e.g., DFS) have been added.
8. Ultimately, **consumer acceptability** should be ensured.
9. **Long term (6–9-mo), well-controlled, randomized efficacy studies** are needed to demonstrate that the fortified food is able to improve iron status under ideal conditions when women of childbearing age or children are fed the iron-fortified food daily in defined amounts. This is needed to confirm the design of the fortified food, especially the fortification compound and the estimated fortification level.
10. **Noncontrolled effectiveness studies** are needed to ensure that the fortification program, containing the efficacious fortified food, is able to maintain and improve adequate iron status in at-risk population groups. These studies are in real-life settings within a defined delivery/distribution system and depend on other factors such as price, consumer accessibility, and consumer acceptability. These studies assume that the fortified foods leave the factory with the correct level of fortification.

The following section summarizes, for each major fortification vehicle, the fortification technology and the iron fortification compounds currently used, and the research and development that was needed to achieve efficacy while avoiding adverse sensory changes.

### Wheat and maize flours

Wheat and maize millers use similar processes with similar equipment (54). Both wheat and maize flours have been fortified for several decades and thus the fortification process for both flours is well established and relatively straightforward, because both the food vehicle and micronutrient premix are in powder form. During the fortification process, a micronutrient premix is precisely added via a feeder directly onto the milled flour on a moving conveyer and then mixed for homogeneity (54). The selection of sufficiently bioavailable iron compounds that cause no sensory changes, however, needed considerable development. Barrett and Ranum (55) summarize the iron compounds tested in wheat and other cereal flours and describe the sensory and technical problems encountered. The main sensory problem with cereal flours was iron-catalyzed oxidative rancidity, which could be provoked by soluble iron compounds such as ferrous sulfate, particularly during longer storage of flours under hot, humid conditions. Unacceptable color reactions when fruits were added to the iron-fortified cereal foods were also a risk. The organoleptically inert elemental iron powders initially became the iron fortification compound of choice for cereal flours.

Wheat and maize flours now have clearly established international guidelines for iron fortification (56, 57). The 4 recommended iron fortification compounds are ferrous sulfate, ferrous fumarate, NaFeEDTA, and electrolytic iron powder. The suggested fortification levels were based on sensory studies, efficacy studies in women and children in LMIC, the relative bioavailability of the iron compound as measured by human isotopic studies, and the estimated daily flour intake. For example, for a population with a daily flour consumption of 150–300 g/d, WHO (56) recommends that wheat flour should be fortified with 30 ppm iron as ferrous sulfate or ferrous fumarate, 60 ppm iron as electrolytic iron powder, or 20 ppm iron as NaFeEDTA. Electrolytic iron powder is recommended only for low-extraction flours with a consumption of ≥150 g/d and is not recommended for nixtamalized maize flour (57).

NaFeEDTA is the only WHO-recommended iron compound for high-phytate wholegrain wheat and maize flours (56, 57). Absorption from wholegrain wheat bread rolls fortified with NaFeEDTA was 4 times higher than with ferrous sulfate (27), and NaFeEDTA-fortified wholegrain atta flour used over 7 mo for chapattis in the school meals of Indian children substantially improved their iron status (58). Aaron et al. (59) reported that the cost of the WHO micronutrient premix (iron, zinc, folic acid, vitamin B-12, and vitamin A) for wheat flour fortification was 3-fold higher with NaFeEDTA than with ferrous sulfate, and 2-fold higher with NaFeEDTA than with ferrous fumarate.

### Rice

Because rice is predominantly eaten in the grain form, rice fortification is technically more difficult than with wheat or maize flour. Coating and extrusion are the 2 main technologies for rice fortification and they both require the manufacture of micronutrient-fortified rice kernels that are subsequently blended with nonfortified rice kernels. When using the coating technology, nonfortified rice grains are coated with liquid waxes or gums containing the added micronutrients and then dried prior to blending. With the extrusion technology, rice flour is blended with a powdered micronutrient premix and fortified kernels are extruded to a similar shape and appearance as the nonfortified rice kernels with which they are subsequently blended. With both procedures, the fortified kernels are typically blended at 0.5–2% with nonfortified rice. The precise blending ratio depends on the desired nutrient concentrations and the amounts of the different micronutrients that have been added to the fortified kernels. Micronutrients are more evenly distributed in extruded grains than in coated grains, and extruded grains are less vulnerable to micronutrient losses during preparation methods such as rinsing, soaking, and cooking in excess water.

A third technology for rice fortification is dusting, where a micronutrient premix is simply applied to rice kernels. Although rice has been fortified in the United States for many years with this technique, dusting has not been introduced globally because the micronutrients are mostly lost if the rice grains are rinsed before cooking or lost when the cooking water is discarded.

With the development of the extruded fortified kernel technology, rice fortification has gathered momentum in recent years (40). Extrusion can be carried out at different temperatures (cold, warm, and hot) (60). Warm and hot extrusion partially or completely gelatinizes the starch so that it holds the kernel together and increases its transparency and sheen so as to more closely resemble nonfortified rice grains that are similarly transparent. Cold extrusion requires a binder to hold the kernel together and, like warm extrusion, can be made with special pasta presses. Hot extrusion requires more expensive, single- or twin-screw extruders and more capital investment.

Extruded kernels are, however, extremely sensitive to color changes with added iron, and FPP is the only iron compound that causes no color change (43). During the development process, FPP was mostly added as either micronized ground ferric pyrophosphate (MGFP; 2.5-μm particle size) or micronized dispersible ferric pyrophosphate (MDFP; 0.3-μm), although in a recent study isotopically labeled FPP, similar to the regular, commercial FPP, was used (61). Regular FPP (as used in infant foods) has a particle size of about 20 μm. MDFP is better absorbed than regular FPP, but relative absorption in humans depends on the food vehicle (9). In rat studies, there was no evidence that the 2.5 μm particle sized MGFP is better absorbed than regular FPP (62).

A recent development in rice fortification is the discovery of a novel enhancer of iron absorption for FPP. When trisodium citrate and citric acid were added with isotopically labeled FPP during rice extrusion, iron absorption from FPP in humans almost doubled to a level similar to that from ferrous sulfate, and it was suggested that the hot extrusion process transformed the insoluble FPP into more soluble FPP citrate complexes (61). There were no reported color changes.

### Milk products

No special equipment is needed to fortify milk with micronutrients. The fat-soluble vitamins are first homogenized with an aliquot of milk in a premix, whereas the water-soluble minerals and vitamins are added directly, either manually or by metered addition. The fortified milk is subsequently agitated, pasteurized, homogenized, and heat treated before packaging. Dried milk can be fortified either prior to or after spray drying.

Reconstituted dried cow milk can be a useful fortification vehicle to provide iron to young children. It is, however, a modest inhibitor of iron absorption due to relatively high concentrations of calcium (63) and casein (64). The addition of ascorbic acid to commercial powdered milk formulas (65) or to dried cow milk fortified with ferrous sulfate or ferrous gluconate is common practice and can overcome the inhibition of the milk constituents and improve iron absorption and efficacy in young children. A 2:1 molar ratio of ascorbic acid to iron is recommended (39), although this is reported to increase the cost of iron fortification with ferrous sulfate ∼15-fold (66).

The combination of soluble iron compounds and ascorbic acid, however, causes unacceptable flavor changes to liquid milk; consequently, iron fortification of liquid milk has not been widely practiced. WHO has recommended FBG, MDFP, and ferric ammonium citrate for liquid milk fortification (39). FBG is sensorily acceptable in liquid milk and additionally partly overcomes milk's inhibition of iron absorption. However, the use of this patented compound has been limited because of its high cost (52).

### Salt

Until recently, the primary use of salt as a vehicle for fortification has been with iodine and, over the last 20 y there has been a considerable expansion of salt iodization in combination with a modernization of salt refining (67). The iodization process has been integrated into the existing production or refining lines using 2 different procedures. In the “wet” method, a solution of potassium iodate is dripped or sprayed onto the salt at a uniform rate as it is transported on a conveyer belt. In the “dry” method, potassium iodate or iodide powder is first mixed with either dry salt, or a filler such as calcium carbonate, and the mixture is then added to dry salt in a batch or continuous blender (67). Many countries have modern salt production facilities, but in many LMIC, there remain small and medium-sized producers using far less sophisticated iodization methods. To manufacture DFS, the selected iron formulation is added to the dry iodized salt in a batch or continuous blender. Producing DFS requires several stages of blending and probably cannot be produced in small- or medium-scale facilities.

On reviewing the DFS literature, Baxter and Zlotkin (68) identified 5 major iron formulations for DFS. They classified Type 1 as with ferrous fumarate, either nonencapsulated (Type 1a), encapsulated (fluidized bed agglomeration) (Type 1b), or encapsulated (extrusion agglomeration) (Type 1c); Type 2 as with ferrous sulfate plus SHMP; Type 3 as with ferrous sulfate plus SHMP, malic acid, and sodium dihydrogen phosphate; Type 4 as with ferrous sulfate encapsulated with partially hydrogenated vegetable oil; and Type 5 as with MGFP. When possible, this nomenclature has been used in the articles that follow this introduction.

Salt is more difficult to fortify with iron than cereals or milk because there is a wide variation in the quality of raw salt produced in terms of purity, moisture content, and particle size. Some common salts, with high impurities and moisture, are extremely sensitive to the formation of unacceptable color changes on addition of iron, and additionally iron can lead to iodine losses during storage by catalyzing the oxidation of iodate or iodide to iodine gas (69). Under unfavorable conditions, iodine losses can be almost complete (69). Another concern, which has not been well investigated, is that iron in DFS could change the color of foods to which DFS is added, especially if meals or foods contain vegetables high in polyphenol compounds.

The development of DFS has taken place over >40 y, primarily in India, firstly by the National Institute of Nutrition in Hyderabad, and subsequently by NI and the Swiss Institute of Technology (ETH Zurich). In the early, extensive Indian trials on DFS (70), various iron compounds, iron complexing agents (to prevent color formation and/or iodine loss), and iron absorption promotors were investigated. Ferrous sulfate and other soluble iron compounds, including NaFeEDTA, rapidly turned the salt brown and accelerated iodine losses. FPP and ferric orthophosphate (FOP) caused no adverse color reactions or iodine losses for several months under a variety of storage conditions but, at that time, were considered unsuitable for salt fortification because of their lower absorption. SHMP was an effective complexing agent and prevented adverse color formation with ferrous sulfate and other soluble iron compounds.

Narasinga Rao (71) subsequently proposed ferrous sulfate plus SHMP (DFS Type 2) as the best approach for DFS. The SHMP prevented color formation and greatly decreased iodine losses with no decrease in iron absorption. Several large efficacy studies were undertaken but iron efficacy of this combination could not be convincingly confirmed, largely due to the use of Hb alone to monitor iron status. Repeating these feeding studies with biomarkers specific to iron status, and taking account of the inflammation status of the subjects, would be expected to show good efficacy of ferrous sulfate plus SHMP. Nevertheless, the failure to demonstrate efficacy, and the need for high-quality dry salt, led to the development of a more sophisticated EFF by NI, and to further studies with FPP added at a higher fortification level and ground to a smaller particle size in an attempt to improve bioavailability. Although good efficacy has been reported for both approaches, further improvements are still possible and, more importantly, further improvements are still needed with respect to the prevention of adverse color formation and iron-catalyzed iodine losses.

The EFF developed by University of Toronto and NI (used in DFS Type 1b or 1c) for addition to iodized salt is more sophisticated than the encapsulated ferrous fumarate or encapsulated ferrous sulfate commonly used in the food industry (9), which is usually coated with partially hydrogenated soybean oil, making up ∼50% of the compound. The composition of NI EFF, and its manufacturing process, have evolved during its development. The original NI EFF used in DFS Type 1b included soy stearine, titanium dioxide (to mask the red color of ferrous fumarate), HPMC, and SHMP, and represented ∼55% of the compound. It was manufactured by a granulation process followed by a coating process. During the granulation process, a mixture of ferrous fumarate, water, HPMC, SHMP, and titanium dioxide were agglomerated on a fluidized bed dryer into granules of similar size to salt grains. During the subsequent coating procedure, microencapsulation technology was used to coat the agglomerated ferrous fumarate granules with a suspension of titanium dioxide in soy stearine (68).

However, because of sensory concerns, including the appearance of black specks in the salt and a tendency for some batches of EFF granules to float on water (72), a new manufacturing process has recently been developed for EFF, which is now used in DFS Type 1c, using the extrusion process. The new manufacturing process is still evolving and consists of the preparation of a ferrous fumarate dough with an edible flour, water, and vegetable oil. The dough is extruded through a fine pasta die, cutting the strands to size, coating with titanium dioxide, and microencapsulating by spraying with HPMC and soy stearine (73). This evolving landscape for the manufacturing process and composition of extruded EFF poses limitations to the evaluation of this technology at the present time.

Studies evaluating adverse color formation and increased iron-catalyzed iodine losses in DFS, with both EFF and MGFP, have been inconsistent, but indicate that color formation and extensive iodine losses can occur when lower quality salt is used in DFS and stored in hot humid climates (44, 74). The need for further developments with DFS Type 1b and Type 5 to avoid color changes and iodine losses is thus clear and has led recently to a new manufacturing process used in DFSType 1c.

In relation to efficacy and iron absorption, there is a need to know whether the extrusion and encapsulation processes used to manufacture the EFF used in DFS Type 1c influence iron absorption from ferrous fumarate in humans. It would also be important to further investigate the potential for iron absorption enhancers in DFS. Ascorbic acid was reported to cause an unacceptable pink coloration (70) and would not be stable during storage, but further studies on the influence of SHMP on iron absorption from ferrous sulfate, EFF, and FPP could provide useful information. There are also several enhancers of iron absorption that might increase absorption specifically from FPP. These include the addition of tetra sodium pyrophosphate (75), trisodium citrate, and citric acid (61) and the addition of sodium hydrogen sulfate, which was reported to increase iron absorption from FOP in human subjects (76). There are currently research efforts targeted at improving the iron compounds used in DFS by utilizing enhancers, and testing the bioavailability of new or existing DFS formulations in use (**Textbox 2**).

Textbox 2 In-progress DFS research.

Ferric pyrophosphate (FPP) is widely used to fortify commercial infant cereals that are sensitive to color changes and has demonstrated good efficacy in young children (9). Micronized FPP is currently being used to fortify rice and has been extensively evaluated in research studies for DFS fortification (Type 5 DFS). The main benefit of FPP is that it does not cause color reactions in foods that are sensitive to color changes with added iron. Its white color also makes it more easily blended into light-colored foods such as refined salt. Its disadvantages are that it is water-insoluble, and that it has a lower relative bioavailability than other major iron fortification compounds, so needs a higher fortification level. A further disadvantage of FPP is that, when ground to form MGFP, it has been reported to cause high iodine losses in moist salt.

Given the importance of a formulation that avoids color changes in storage as well as in foods cooked with DFS, there are research efforts underway to reassess the potential of regular FPP as an iron compound for DFS and compare its bioavailability with the current Type 1c DFS using encapsulated ferrous fumarate (M Zimmermann, ETH Zurich, personal communication, 2019).

Additionally, enhancers to improve iron absorption from FPP will be tested in an attempt to improve its bioavailability. These include citric acid and trisodium citrate (which have increased iron bioavailability from FPP in fortified rice) and sodium pyrophosphate (which has increased iron bioavailability from FPP in bouillon). The FPP and added enhancers will be encapsulated to minimize iodine losses. The enhanced FPP formulations will be tested for color stability and iodine retention in DFS storage studies, as well as bioavailability.

## Estimating the Iron Fortification Level for National Fortification Programs

The theoretical way to fortify staple foods and condiments with micronutrients is to add the micronutrient to ≥1 food vehicles so as to bring the intake of the micronutrient above its estimated average requirement (EAR) in 97.5% of the most at-risk population group (39). This method, however, cannot be used for iron because iron intakes of menstruating women and children are not distributed normally. To estimate iron fortification levels, tables of the “probability of inadequacy of dietary iron intakes” in women and children consuming different amounts of iron have been published by WHO (39). The amount of additional iron intake needed to decrease the probability of iron inadequacy in women and children to <2–3% can be calculated from these tables. The additional iron intake needed is then adjusted if necessary based on the relative bioavailability of the iron fortification compound, and the iron fortification level is calculated based on the consumption pattern of the fortification vehicle.

The above approach is recommended when nationally representative dietary intake data have been collected. Such data are usually not available in LMIC, and a more pragmatic, evidence-based approach was used to define iron fortification levels for wheat and maize flour (56). After reviewing a series of efficacy studies in women and children from LMIC consuming different iron-fortified staple foods and condiments, it was estimated that efficacy of an iron fortification program could be achieved by providing a minimum additional amount of 7 mg Fe/d as ferrous sulfate (3). This amount represents ∼50% of the EAR for both women of reproductive age and 14–18-y-old boys and girls who are consuming a 10% iron bioavailability diet (9) (**Table 1**). These population groups have the highest iron requirements and are the most at risk.

**TABLE 1:** Estimated average requirement (EAR) of iron for women, adolescents, and children consuming a diet with 10% bioavailable iron^1^

Sex/age category	EAR, mg Fe/d
Women 18–50 y	14.4
Boys 14–18 y	13.9
Girls 14–18 y	14.2
Boys 9–13 y	10.6
Girls 9–13 y	10.3
Children 4–8 y	7.4

1 Recalculated from: Institute of Medicine. *Dietary Reference Intakes for vitamin A, vitamin K, arsenic, boron, chromium, copper, iodine, iron, manganese, molybdenum, nickel, silicon, vanadium and zinc*.Washington (DC): National Academies Press; 2001.

Other soluble iron compounds, and ferrous fumarate, are as well absorbed as ferrous sulfate and should also be added to provide 7 mg Fe/d. Because of their estimated 50% lower relative bioavailability, FPP and electrolytic iron powder should be added to provide 14 mg Fe/d. Assuming NaFeEDTA is about twice as well absorbed as ferrous sulfate from phytic acid–containing foods, 3.5 mg Fe/d should be added; however, the lowest level of NaFeEDTA that has so far shown iron efficacy in feeding studies is 4.6 mg Fe/d, and this value was recommended (3).

In relation to iron fortification of DFS or rice with FPP or MGFP, the recommendation would be to provide 14 mg Fe/d. For DFS Type 1c, fortified with EFF, the recommendation would be to provide 7 mg Fe/d, assuming that the extrusion of ferrous fumarate with cereal flour and encapsulation do not influence iron absorption. Because the effect of the encapsulation is unknown, the influence of the current extruded EFF capsule on iron absorption from ferrous fumarate in humans should be measured. There is little information to provide a recommendation for FBG in milk products but, assuming a 2-fold better iron absorption than from ferrous sulfate, the recommendation would be to provide 3.5 mg Fe/d as FBG.

## Monitoring the Impact of Iron Fortification

Iron-fortified foods fed in large-scale programs or in well-controlled efficacy studies are judged to have demonstrated impact if the iron status of the study population is significantly increased over the feeding period. As explained earlier, iron status cannot be monitored by Hb alone and must be monitored by specific iron status biomarkers. However, an additional concern is the presence of widespread infections and inflammation in the study population. Iron status must be evaluated differently depending on whether or not the study population is affected by inflammation. In the absence of inflammation, Hb, serum ferritin (SF), transferrin receptor (TfR), zinc protoporphyrin (ZPP), and increased body iron stores based on SF and TfR, are all considered useful biomarkers of iron status (16). In the presence of infection, SF and TfR can still be used but they must first be corrected for the effects of inflammation (77, 78).

Infections and inflammation are common in tropical areas of Asia, Africa, Latin America, and the Caribbean. In these areas, inflammation has a major influence on iron metabolism, and on the biomarkers of iron status, complicating the measurement of iron status, and making the impact of iron fortification programs difficult to demonstrate. Inflammation causes the liver to increase hepcidin production. Hepcidin then degrades the iron transporter ferroportin, and restricts the passage of iron into the plasma. The most important effect is a decrease in the recycling of red cell iron stored in the macrophages of the reticuloendothelial system. The recycling of iron from senescent red cells usually provides ∼20 mg Fe/d. The passage of iron into the plasma from the intestinal cells is also restricted by hepcidin and iron absorption is decreased (79). However, it is the inflammation-induced decrease in the recycling of red cell iron that has the greatest impact on iron status, because iron recycling provides 10–20 times more daily iron than does dietary iron absorption (38). Iron fortification interventions are understandably less effective in the presence of infections and inflammation.

The monitoring of iron status in areas of widespread infection and inflammation is further complicated because the biomarkers of iron status are also influenced by the inflammation. SF, a biomarker of liver iron stores, is perhaps the most widely used biomarker of iron status. However, it is also an acute-phase protein that increases with inflammation. In order to be a useful iron status measure in these circumstances, the SF values must first be adjusted for inflammation (77). A correction for inflammation also exists for TfR (78).

Recently, Ganz (80) proposed a hypothesis that could have a major influence on the measurement of iron status in the presence of infections and inflammation. He suggested that when iron supply to the body is severely restricted, as with the prevention of iron recycling during infection and inflammation, red cell production is curtailed so that the extremely low iron supply can be preferentially utilized for the essential enzymes in the tissues (e.g., in the brain). This means that using red cell parameters such as ZPP and TfR to monitor a subject's iron status in the presence of inflammation could underestimate iron status in the tissues (38). If the Ganz hypothesis is confirmed, the only useful iron status biomarker to measure the impact of iron fortification programs in areas of infection and inflammation could be the adjusted SF.

The safety of iron interventions in malaria endemic areas is also an issue because iron supplements, especially when given without food, can increase the severity of the malarial infections (81). The likely explanation is that the rate of iron influx into the plasma from high-dose oral supplements exceeds the rate of iron binding to transferrin and a quantity of non–transferrin-bound iron (NTBI) is formed (82). It is proposed that NTBI increases the intensity of malarial infections by increasing the sequestration of malaria-infected RBCs in the capillaries of the brain and intestine, causing cerebral malaria and further increasing the permeability of the intestinal barrier to the passage of pathogens. At the same time, high iron doses stimulate the growth of pathogenic bacteria in the stool, increasing the potential for bacteremia. The normal immune response to malaria, as well as other infections and inflammatory disorders, is to prevent further microbial growth by stimulating hepcidin synthesis and preventing the passage of iron into the plasma.

Unlike with iron supplements, little or no NTBI is formed on consumption of iron-fortified foods, even when the foods are fortified with ferrous sulfate (83), and there is no evidence that iron-fortified foods increase the intensity of malaria or other infections. Iron fortification compounds that are slowly absorbed, such as FPP, produce even less NTBI than soluble iron compounds (9, 84). Iron-fortified foods, however, can increase the number of pathogens in the lower gut to the detriment of the beneficial barrier bacteria (85). Nevertheless, although Gera et al. (8) reported that oral iron supplements caused an 11% increase in diarrhea in children in developing countries, they noted that there was a near absence of effect with iron-fortified foods. They suggested that this was because the lower amount of iron added to fortified foods is closer to the physiological situation.

## The Way Forward

Salt, wheat flour, maize flour, rice, and milk are the 5 major staple foods or condiments that have been most utilized as vehicles for micronutrient fortification at the global level. All have been used as a national public health strategy to target ID or to target deficiencies in other micronutrients. By far the most successful intervention has been the iodine fortification of salt. Salt iodization facilities have been installed in >140 countries and ∼5 billion people, 75% of the global population, have access to iodized salt (67). As a result, goiter, cretinism, and severe iodine deficiency have been eradicated from many countries. In comparison, and for reasons discussed earlier, the iron fortification of wheat and maize flours has made only a modest impact on anemia in LMIC, especially when compared with the marked decrease in neural tube defects when cereal flours and other cereal foods are fortified with folic acid.

There are several reasons for the global success of salt iodization. The most important is that salt, unlike wheat, rice, or maize, is universally consumed in predictable amounts by all population groups in all countries worldwide. Iodine also has a relatively low EAR of ∼100 μg/d in adults and children, which is easily carried by an average salt consumption of 5–10 g/d without causing changes to the color of the salt or to foods cooked with iodized salt. Iodine absorption is also little influenced by other dietary components.

Iron fortification of foods is frequently needed as a public health strategy to combat ID and IDA in many countries worldwide. Iron-fortified foods will improve iron status provided the iron fortification compound is chosen wisely and fortification level estimated according to the amount of vehicle consumed, the estimated dietary iron bioavailability, and the relative absorption of the fortification compound. The choice of iron fortification vehicle for national programs until now has largely been based on consumption patterns of wheat flour, maize flour, and rice, and to a lesser extent on the degree of industrialization of the respective cereal industries. Dried milk powder has been the preferred food vehicle to provide additional iron to infants and young children.

Based on consumption patterns, however, DFS has the potential to be the universal global carrier for both iodine and iron provided that the technical challenges for the iron fortification of salt can be overcome. The following articles in this series discuss to what extent we are ready to manufacture a DFS that has the potential to make a significant contribution to reduction in ID in many national contexts.

## Notes

This work was supported by the Iodine Global Network.

Publication costs for this supplement were defrayed in part by the payment of page charges. The opinions expressed in this publication are those of the authors and are not attributable to the sponsors or the publisher, Editor, or Editorial Board of *The Journal of Nutrition*.

Author disclosures: The author reports no conflicts of interest.

Abbreviations used: DFS, double-fortified salt; EAR, estimated average requirement; EFF, encapsulated ferrous fumarate; FBG, ferrous bisglycinate; FOP; ferric orthophosphate; FPP, ferric pyrophosphate; Hb hemoglobin; HMPC, hydroxy propyl methyl cellulose; ID, iron deficiency; IDA, iron deficiency anemia; LMIC, low- and middle-income countries; MDFP, micronized dispersible ferric pyrophosphate; MGFP, micronized ground ferric pyrophosphate; NaFeEDTA sodium iron ethylenediaminetetraacetic acid; NI, Nutrition International; NTBI, non–transferrin-bound iron; SF, serum ferritin; SHMP, sodium hexametaphosphate; TfR, transferrin receptor; ZPP, zinc protoporphyrin.
